# Accurately Assessing the Risk of Schizophrenia Conferred by Rare Copy-Number Variation Affecting Genes with Brain Function

**DOI:** 10.1371/journal.pgen.1001097

**Published:** 2010-09-09

**Authors:** Soumya Raychaudhuri, Joshua M. Korn, Steven A. McCarroll, The International Schizophrenia Consortium, David Altshuler, Pamela Sklar, Shaun Purcell, Mark J. Daly

**Affiliations:** 1 Program in Medical and Population Genetics, Broad Institute, Cambridge, Massachusetts, United States of America; 2 Center for Human Genetic Research, Massachusetts General Hospital, Boston, Massachusetts, United States of America; 3 Division of Rheumatology, Immunology, and Allergy, Brigham and Women's Hospital, Boston, Massachusetts, United States of America; 4 Graduate Program in Biophysics, Harvard University, Cambridge, Massachusetts, United States of America; 5 Department of Genetics, Harvard Medical School, Boston, Massachusetts, United States of America; 6 Department of Molecular Biology, Massachusetts General Hospital, Boston, Massachusetts, United States of America; 7 Department of Psychiatry, Massachusetts General Hospital, Boston, Massachusetts, United States of America; 8 Psychiatric and Neurodevelopmental Genetics Unit, Massachusetts General Hospital, Boston, Massachusetts, United States of America; 9 Stanley Center for Psychiatric Research, Broad Institute, Cambridge, Massachusetts, United States of America; University of Alabama at Birmingham, United States of America

## Abstract

Investigators have linked rare copy number variation (CNVs) to neuropsychiatric diseases, such as schizophrenia. One hypothesis is that CNV events cause disease by affecting genes with specific brain functions. Under these circumstances, we expect that CNV events in cases should impact brain-function genes more frequently than those events in controls. Previous publications have applied “pathway” analyses to genes within neuropsychiatric case CNVs to show enrichment for brain-functions. While such analyses have been suggestive, they often have not rigorously compared the rates of CNVs impacting genes with brain function in cases to controls, and therefore do not address important confounders such as the large size of brain genes and overall differences in rates and sizes of CNVs. To demonstrate the potential impact of confounders, we genotyped rare CNV events in 2,415 *unaffected* controls with Affymetrix 6.0; we then applied standard pathway analyses using four sets of brain-function genes and observed an apparently highly significant enrichment for each set. The enrichment is simply driven by the large size of brain-function genes. Instead, we propose a case-control statistical test, *cnv-enrichment-test*, to compare the rate of CNVs impacting specific gene sets in cases versus controls. With simulations, we demonstrate that *cnv-enrichment-test* is robust to case-control differences in CNV size, CNV rate, and systematic differences in gene size. Finally, we apply *cnv-enrichment-test* to rare CNV events published by the International Schizophrenia Consortium (ISC). This approach reveals nominal evidence of case-association in *neuronal-activity* and the *learning* gene sets, but not the other two examined gene sets. The *neuronal-activity* genes have been associated in a separate set of schizophrenia cases and controls; however, testing in independent samples is necessary to definitively confirm this association. Our method is implemented in the PLINK software package.

## Author Summary

Specific rare deletion and duplication events in the genome have now been shown to be associated with neuropsychiatric diseases such as 16p11.2 to autism and 22q11.21 to schizophrenia. However, controversy remains as to whether rare events impacting certain pathways as a group increase the risk of disease, and if so, what those pathways are. Other studies have used standard gene-set enrichment approaches to demonstrate that events discovered in cases contain more genes in neuro-developmental pathways than would be expected by chance. However, these analyses do not explicitly compare the relative enrichment in cases to any enrichment that may also be present in controls. Therefore, they can be confounded by the large size of brain genes or by larger size or frequency of CNVs in cases. Here we propose a case-control statistical test to assess whether a key pathway is differentially impacted by CNVs in cases compared to controls. Our approach is robust to skewed gene sizes and case-control differences in CNV rate and size.

## Introduction

Multiple recent studies have demonstrated a convincing and statistically significant excess of rare CNVs in individuals affected by schizophrenia compared to unaffected individuals [1]–[4]. Similar observations have now been made separately in autism [5]–[7] and bipolar disorder [8]. However, it is typically not readily evident which individual CNV events are pathogenic since (1) many rare events are seen in the general population and the excess in cases is relatively modest and (2) individual events are too rare to demonstrate definitive association in realistically sized patient collections. Hence, it is challenging to translate these rare CNV events into a clear understanding of disease pathology. To identify candidate genes for follow-up, investigators have employed statistical tests of gene set enrichment, originally developed as an effective approach to interpret gene expression data [9]. Practically, these analyses identify functional gene sets or ‘pathways’ that are over-represented among those genes affected by case CNVs compared to unaffected genes [1], [8], [10], [11], often relying on online resources such as Panther [12], Ingenuity Pathway Analysis (Ingenuity Systems, www.ingenuity.com), and Gene Ontology [13].

For example, gene set enrichment analyses by Walsh *et al.* suggested that rare CNVs in schizophrenia preferentially disrupt those genes with neuro-developmental functions [1]; Zhang *et al.* similarly reported that rare CNVs in bipolar disorder preferentially overlap genes involved in behavior and learning [8]. More recently Glessner *et al.* reported that genes affected by rare and common CNVs in autism are also involved in brain function [11]. While these initial results are highly promising, the gene set enrichment statistical framework as applied to copy number variation is critically limited and potentially confounded.

The key analytical question in this setting is whether an event that impacts a set of genes or a pathway, increases disease risk compared to events that do not impact that pathway. Under the hypothesis that events affecting a specific brain-function pathway are pathogenic, the rate of those events affecting the brain-function pathway should indeed be greater in cases than in controls – ideally fully explaining the observed genome-wide differences in case and control event rates. An alternative possibility is that the increased rate and size of CNVs in cases represents a mutational syndrome or genomic instability, and that they are not in themselves pathogenic. Under that possibility, case events should not preferentially impact any particular gene set; however, differences is size and rate might be observed.

The commonly used gene set enrichment analytical approach used to address this question falls short on two accounts: (1) they do not rigorously compare case event rates to control event rates and (2) since they examine affected genes rather than events, they do not accurately account for the fact that multiple genes might be contributed from a single event or that single genes may be affected by multiple events. Here we propose a straight forward statistical test to explicitly compare the rate of CNVs impacting a specific gene set in cases to the rate in controls that carefully accounts for background differences in CNV rate and size.

A possible consequence of not rigorously comparing event rates in cases to controls is that sets consisting of genes that are more frequently affected by CNVs might spuriously appear to be highly enriched in cases, but also will be highly enriched in controls. Examples of such genes include large genes spanning a massive portion of the genome or those whose functions are highly redundant or non-essential. This is a particularly important issue for neuropsychiatric disease considering the reportedly large size of genes with brain function. Multiple studies of CNVs in the general population have reported enrichment for neuro-physiological genes [14], [15] – suggesting that brain-function genes may be susceptible to CNV events in general, possibly due to their large size or other factors. In fact, published events in neuropsychiatric disease studies often implicate large genes (see Table S1). Others have already noted that gene size itself can bias pathway enrichment analyses in other contexts, such as annotating non-coding elements for function [16], [17]. In particular, Taher et al noted that randomly selected positions in the genome are enriched for proximity to genes involved in “development”, “cell-adhesion”, and “nervous system development” [17]. Some of the published disease studies attempted to address this issue indirectly by applying similar analyses to control events and note the lack of statistical evidence of enrichment for brain function gene sets [1], [8]; however, control events are typically fewer and smaller, implicating many fewer genes, and therefore simply comparing the statistical significance of gene set enrichment results in cases and controls is not adequate.

One possible consequence of examining genes rather than the events they occur in is that individual large events contribute many genes and may skew the analysis much more than smaller events, and cause spurious findings. This is of particular concern since genes with common function can often cluster together on the genome and a single event in one individual affecting a cluster of related genes can naively appear to implicate an entire pathway [18], [19]. One interesting example is the reported enrichment of *psychological disorder* genes in the Zhang et al data set (see Table S1); 11 of the 16 deleted *psychological disorder* genes are in the 22q11.21 region observed in two individuals in the data set [8]. These genes are possibly annotated as psychological disorder genes since rare deletions in 22q11.21 have long been observed among schizophrenia cases [2]. Removal of the two individuals with 22q11.21 events eliminates any enrichment for the *psychological disorder* gene set – suggesting that there is little evidence that this particular set is necessarily relevant to disease outside of the 22q11.21 region. Of course at least one gene in this region is pathogenic, but it is unlikely that >10 in this region are and that in aggregate define a key pathogenic set.

A second possible consequence of examining genes and not the events they occur in is that genes affected in both cases and controls, but at different rates, are not properly accounted for. For instance, a critical gene affected by many pathogenic events contributes equally to a gene set enrichment analyses as a gene sporadically affected by a single event. One interesting example is the *NRXN1* gene, a large gene that plays an important role in synaptic development [20]. Since CNV events affecting *NRXN1* have been observed in both schizophrenic cases and controls, they would contribute equally to a pathway analysis of case events as they would to one of control events. However, the rate of functional events observed in cases is significantly more than in controls; pathway-based approaches could be bolstered if methods explicitly take into account these differences between cases and controls event rates for genes of interest.

Here, we describe a case-control statistical test, *cnv-enrichment-test*, to explicitly compare the rate of CNVs impacting specific genes sets in cases to controls. We show how *cnv-enrichment-test* is robust to even extreme biases in gene size and case-control differences in CNV rate and size. We also demonstrate how standard gene set enrichment approaches is often confounded under realistic scenarios, by gene size and other gene structural features; we demonstrate these confounders in a set of 2,415 controls genotyped for rare single-event deletions. We finally apply the *cnv-enrichment-test* to examine genes with brain function within a large dataset of CNVs identified in schizophrenia cases and controls published by the International Schizophrenia Consortium (ISC) [2] and demonstrate nominal evidence of association for previously described gene sets.

## Results and Discussion

### Standard enrichment analysis to test gene sets affected by rare CNVs

Set enrichment is the standard approach to test whether genes impacted by CNVs in cases affect specific pathways. Specifically, the overlap between the set of genes affected by CNVs is compared to the set of genes with a particular function.

Genes affected by a CNV might be defined as *disrupted* genes or *overlapped* genes. *Disrupted* genes are those genes that have a CNV boundary that falls within the boundaries of its transcript [1]. *Overlapped* genes are a superset of those genes whose transcripts are either disrupted by a CNV or are fully contained by a CNV [8]. Since genes rarely overlap each other, a single CNV event might contribute up to two *disrupted* genes but many *overlapped* genes. Both have been previously examined in the literature. Unless otherwise specified, this study emphasizes overlapped genes.

After identifying the genes affected (overlapped or disrupted) by a CNV, we then identify genes with a specific process or within a specific pathway. We apply a two-tailed Fisher's test to assess whether the number of affected pathway genes is statistically significantly different than might be affected by chance. The critical assumption in gene set-based analyses is that there is a single independent observation per gene, not connected to the gene's size or structural features.

### A case-control framework to test gene sets affected by rare CNVs

As an alternative, we propose a simple case-control strategy to test gene sets or pathways for association to disease: the “*cnv-enrichment-test*”. This strategy is consistent with the case-control association framework used in CNV and SNP disease association studies [21], [22]. A direct case-control comparison avoids any ascertainment bias that might be the consequence of structural features of genes within a set, since the same biases will apply equally to both cases and controls.

We are careful to control for case-control differences in CNV rate and size, since those differences can artificially induce a pathway association. For example, if the rare CNV rate in cases is more frequent or larger than in controls, then on average all genes will be impacted more often in cases, and any arbitrary gene set might appear to be affected more commonly in cases than in controls. Also, if rare CNVs are smaller but more frequent in cases than in controls, then sets of larger genes might appear to be impacted more often in cases than in controls.

To assess whether CNV events specifically overlapping genes in the pathway of interest are enriched in cases compared to controls, we propose the following logistic model:
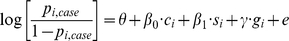
where *p_i_* is the probability that individual *i* is affected, *c_i_* is an integer that indicates the number of rare CNVs that an individual *i* has, *s_i_* is the average size of those events, *g_i_* is the count of gene within a pre-specified gene set affected by a cnv, and *e* is an error term. The terms *θ*, *γ*, *β_0_*, and *β_1_* are logistic regression parameters that are optimally determined to maximize the likelihood of the data. The *θ* term (the intercept) represents the background log likelihood for each individual, *γ* is the increase in log-likelihood per affected gene within the gene set, *β_0_* is the increase in log-likelihood per rare CNV, and *β_1_* is the increase in log likelihood per kilobasepair of average rare CNV size. The *cnv-enrichment-test* simply tests if *γ* is significantly different from 0.

In principle, previous studies in schizophrenia that have shown excess CNVs in affected individuals corresponding to a positive *β_0_*. It has also been demonstrated that individuals with neuropsychiatric disease often have larger events, consistent with a positive *β_1_* term. On the other hand, if there is a “causal” gene set *g*, then adding it to the model should attenuate the magnitude of both *β_0_* and *β_1_* and result in a convincingly positive *γ*. An independent odds ratio estimate, e*^γ^*, can be calculated for the additional increased risk of disease if an event affects a gene in set *g*.

This approach is not confounded by functionally related genes that cluster on the genome. Since risk is estimated on a per individual basis, a single spurious observation will not dramatically impact the statistical significance of any of the parameter estimates. So, a rare single event, which happens to overlap multiple related genes within the gene set that is being tested, will not contribute substantially to the significance of *γ* - even though potentially many genes from that pathway are implicated. Of course, if many such events are observed, with a proclivity towards either cases or controls, then estimates for *γ* might appropriately be more significant.

The approach can be extended to do a meta-analysis if patient data is aggregated, and indicator variables are included to denote the dataset that the patient sample was derived from. Indicator variables would potentially account for specific differences across data sets, such as the proportion of individuals that are cases and also underlying biases in case severity.

This approach can be facilely applied to gene-sets ranging widely in size. It can equally be applied to a single gene, for example to identify whether a gene such as *NRXN1* has more case-events than control-events after controlling for genome-wide differences in CNV size and rate. It can also be easily applied to the set of all genes in the human genome to test if genes in general are more often affected in cases than controls. We caution that in data sets with too few individuals, association to smaller gene sets might be difficult to detect given power limitations; furthermore the asymptotic p-value might be inaccurate. In cases where too few events have been genotyped the asymptotic *p*-value can be replaced by a *p*-value based on robust permutation testing instead.

We have implemented this test in the publicly available genetic data analysis software, PLINK [23].

### CNV-enrichment-test is robust to skewed gene size, even if there are case-control differences between the size and rate of CNVs

**Figure 1:**
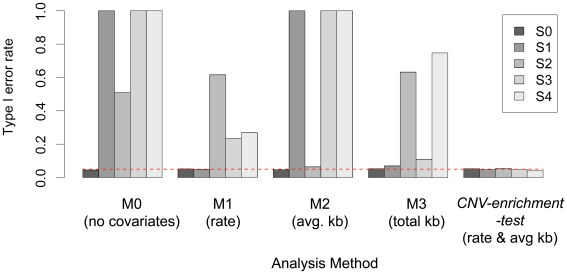
Performance of each of the five proposed models (M0–M4) across five hypothetical scenarios. For each of the scenarios (**S0–S4**) outlined in Table S2 we simulated 10,000 datasets to calculate the type I error rate for the enrichment test, for a nominal rate of 0.05. Only the model **M4**, controlling for CNV rate and average size, obtains an appropriate type I error rate under all scenarios where case-control differences in size and rate are presence. Other models, fail to adequately control for these confounders. The **M4** model is presented in the main text as the *cnv-enrichment-test*.

To demonstrate that the *cnv-enrichment-test* does not detect spurious associations due to gene features that predispose key gene sets towards CNVs, we carefully considered gene size. We created an extreme hypothetical scenario (**S0**, see Table S2). Here, every fifth gene was designated as a hypothetical “brain gene”; brain genes were set to be considerably larger than other genes (50 kb versus 10 kb). For a single hypothetical chromosome, 250 Mb in length, we placed 2000 evenly spaced, non-overlapping genes. In all scenarios we simulated CNV data for 2000 cases and 2000 controls, specifying the mean CNV size at 100 kb (range 10 kb to 150 kb, standard deviation 30 kb) and the CNV rate per individual at 0.25. Reassuringly, in this simulation *cnv-enrichment-test* for “brain-genes” demonstrated *p*<0.05 association in 4.1% of 10,000 simulated datasets, suggesting that it estimates the type I error rate accurately (see Figure 1).

However, in practice, differences between the size and rate of CNVs might be present due to true genetic differences between cases and controls, as demonstrated in neuropsychiatric disease, or technical differences in array intensity or genotyping platform.

Our method must be robust to these differences and must not spuriously identify pathways with large genes as a consequence of these differences. To test for this we created four extreme scenarios (**S1** **–** **S4**, see Table S2). Under **S1**, we dramatically reduced the control rate of CNVs to 0.05/individual while retaining the same rate in cases (0.25/individual). Under **S2**, we fixed the rate at 0.25/individual in both cases and controls, but reduced the mean CNV size in cases (60 kb) compared to controls (100 kb). Under **S3**, we assigned cases the greater rate and mean size (0.25/individual and 100 kb) compared to controls (0.05/individual and 60 kb); this scenario is analogous to schizophrenia where events are larger and more frequent in cases. Under **S4**, we assigned cases had a greater rate, but smaller mean size (0.25/individual and 60 kb) compared to controls (0.05/individual and 100 kb); this scenario might occur if higher quality genotyping is applied in cases only resulting in better ability to detect smaller CNVs than in controls. We found that the proposed method that controlled for both CNV rate and average CNV size was robust under each of these extreme scenarios and for 10,000 simulated datasets demonstrated appropriate type I error rate at *p*<0.05 under all scenarios (see Figure 1).

To illustrate the importance of controlling for CNV rate and size in this setting where a pathway consists of systematically larger genes, we examined more limited models that do not control for either or both the CNV rate and size. All of these models caused inappropriately high type I error rates under at least one of the above scenarios (see Figure 1) and would demonstrate spurious association to “brain genes”. A simple association test (**M0**) that does not account for either for CNV rate or size at all demonstrates higher rates of false associations under all simulated scenarios where there are case-control differences in size and rate of CNVs (**S1** **–** **S4**). Similarly, controlling for differences in rate only (**M1**) demonstrates higher rates of false associations under almost all simulated scenarios, except for **S0** and **S1**. Controlling for differences in size only (**M2**) demonstrates higher rates of false associations under almost all simulated scenarios, except for **S0** and **S3**. Finally, controlling for differences in total CNV burden (**M3**) demonstrates higher rates of false associations under **S3** and **S4** all simulated scenarios.

### Four plausible sets of genes with brain function

To broadly define genes that control brain function, we used a gene expression tissue atlas to define a broad set of 2,531 preferentially *brain-expressed* genes (see **Materials and Methods**). For secondary analyses, we compiled three more sets of general interest to neuropsychiatric disease: (1) 455 *neuronal-activity* genes defined by Panther and highlighted previously in schizophrenia by Walsh *et al* [1], (2) 126 *learning* genes defined by Ingenutiy and highlighted by Zhang *et al* in bipolar disease [8], and (3) 209 *synapse* genes defined by Gene Ontology. The gene sets overlap; 12 genes are in all four sets.

### Application of set enrichment to rare CNVs in controls demonstrates that brain function genes are enriched

To demonstrate some of the limitations associated with standard set enrichment tests to assess critical gene functions examined the aforementioned gene sets in rare CNVs from controls recruited from the general populations. We used Affymetrix 6.0 chips in conjunction with stringent and uniform quality control to genotype 2,415 unaffected individuals (see Table S3 and Table S4) from four separate studies [8], [24]–[26]; hereafter referred to as ‘meta-controls’. We identified 1,054 single event deletions ranging from 20 kb to 1.9 Mb in size. To obtain the most confident calls possible, we focused only on deletions (see **Materials and Methods**) – though including duplications does not substantially impact our results.

Strikingly, many of the genes that are disrupted (and therefore also overlapped) by rare deletions within the meta-controls have been proposed as candidate genes for neuro-developmental diseases including: *GRM5*, *GRM8*, *FHIT*, *OPCML*, *PTPRD*, *NRXN3*, *NRG3*, *CNTNAP2*, *AUTS2*, *CTNNA3*, *DLG2*, *ERBB4*, *PTPRM*, and *NRXN1*. All of these genes are among the largest in the human genome, with transcripts extending from 550 kb to 2.2 Mb of genome. Except for *GRM5* and *PTPRM*, they are all greater than 1 Mb in length. In particular *DLG2*, *ERBB4*, *PTPRM*, and *NRXN1* were disrupted by 12 individual events in our study; Walsh *et al.* highlighted these four genes as potentially pathogenic based on pathway analysis [1].

**Figure 2:**
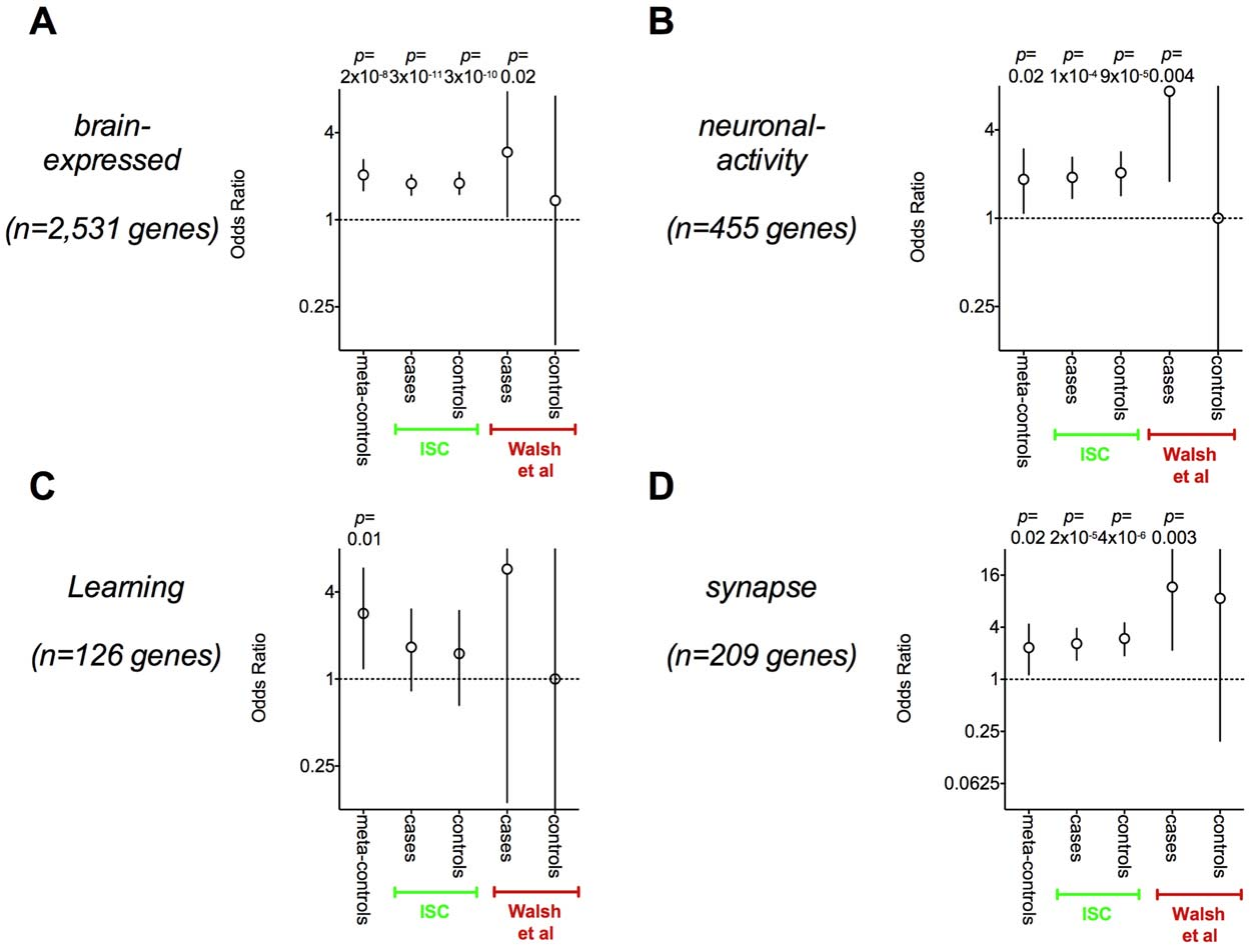
Neurodevelopmental gene sets are enriched in CNVs for affected and unaffected individuals. Here we present results from three gene sets – *neuronal-activity* genes (**A**), *brain-expressed* genes (**B**), *learning* genes (**C**), and *synapse* genes (**D**). For each set we calculate enrichment among genes disrupted by rare CNVs with a Fisher's exact test in meta-controls and also within the affected and unaffected individuals in the Walsh *et al.* study and the ISC study. We explicitly list all *p*-values <0.1. Each point represents an odds ratio and is plotted with a 95% confidence interval. A comparable degree of enrichment was observed across all data sets for each of the gene sets.

As previously observed by Redon et al [14] and Yim et al [15], genes affected by rare CNVs are involved disproportionately in brain function in this control population. The set of genes disrupted by deletions within the meta-controls are enriched for brain-expressed genes (OR = 2.0, *p* = 2×10^−8^) and other brain function gene sets as well (see Figure 2). The enrichment is present, though somewhat less pronounced, if all genes overlapping deletions are included (OR = 1.63, *p* = 4×10^−6^, see Figure 2).

### Gene size confounds gene set enrichment approaches

**Figure 3:**
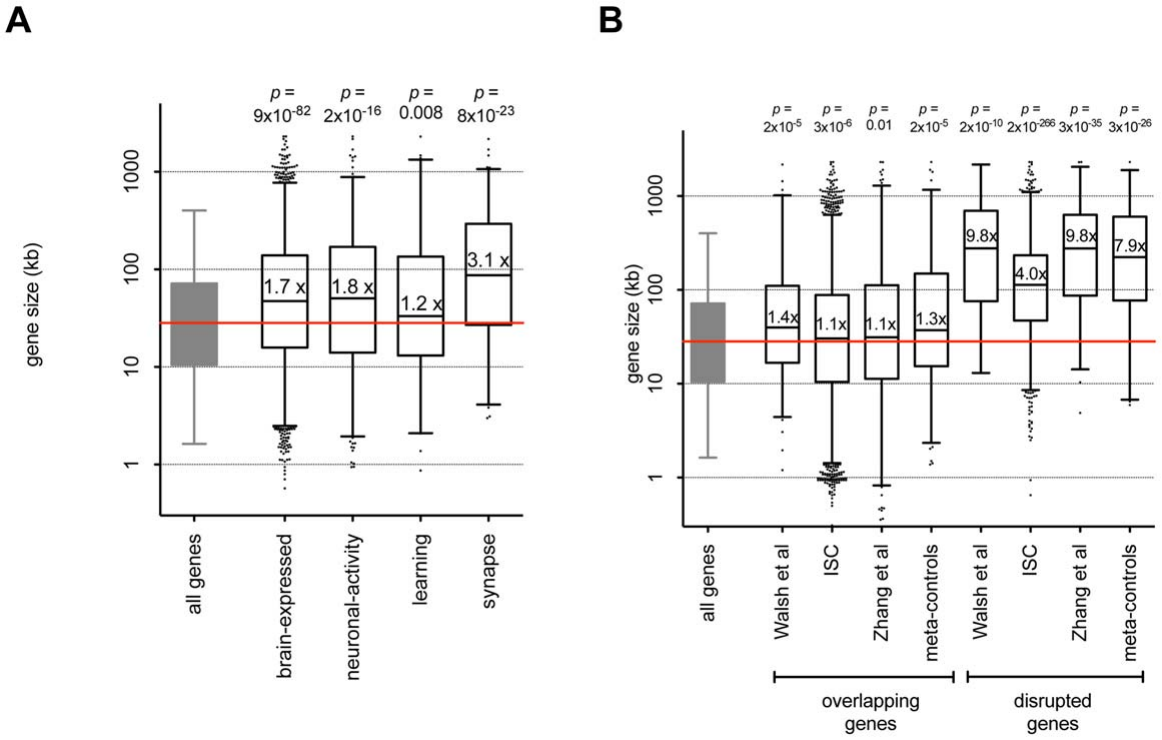
Genes with brain function and genes impacted by CNVs are large. A. Brain-expressed, neuronal-activity, learning, and synapse gene sets consist of large genes. For each gene set we plot the length of each gene, relative to the median length of human genes (28.2 kb). Median gene length is labeled and represented by a horizontal line. Box indicates the range of gene lengths (2.5%–97.5%). Outliers are plotted as dark points outside the box. For each gene set we compared the length of genes within the set and outside the set with a two-tailed rank-sum *p*-value. B. Genes overlapping and disrupted by rare CNVs are large. For each data set we plot the length of genes overlapping (left) and disrupted by (right) rare CNVs, relative to the median length of human genes (28.2 kb). Median gene length is labeled and represented by a horizontal line. Box indicates the range of gene lengths (2.5%–97.5%). Outliers are plotted as dark points outside the box. For each set we compared the length of genes affected and not affected by CNVs with a two-tailed rank-sum *p*-value. To allow for consistent comparisons, we restricted meta-control events to those >100 kb.

To explain this enrichment of rare CNVs affecting brain-function genes in controls, we conjectured that the gene set enrichment approach is confounded by gene size. Three observations support this possibility. First, the transcripts of brain-expressed genes are significantly larger than of other human genes (*p* = 9×10^−82^ by non-parametric rank-sum test, see Figure 3A). The median length of all human gene transcripts is 28.2 kb; in contrast the median length of brain expressed gene transcripts is 47.2 kb (1.7 fold longer). In fact of the genes longer than 1 Mb, 32 out of 48 (67%) are brain-expressed. Genes in the three other gene sets are also significantly longer (1.2–3.1 fold). Second, we note that the genes affected by CNVs are also large. Genes disrupted by events in these meta-controls, as well as previously published data sets by Zhang et al, Walsh et al, and the ISC were large (*p*<2×10^−10^, see Figure 3B). The bias towards large genes is still present, though mitigated, if the analysis is expanded to include all overlapping genes (*p*<0.01, see Figure 3B). Smaller genes *overlapping* a CNV are much more likely to be fully contained by that CNV while larger genes are more likely to extend beyond the boundaries of the CNV and hence be *disrupted* by that CNV. Third, almost all gene ontology [13] (GO) categories consisting of genes with an average size >200 kb are preferentially affected by rare deletions within the meta-controls (see Table S5). These codes implicate functions such as cell adhesion and recognition, neuron recognition, and synaptic pathways.

### Random genomic segments also demonstrate enrichment for brain genes

To quantify the extent to which observed enrichment for these gene sets was simply a consequence of their large size, we tested whether randomly placed genomic segments affect genes with brain function genes preferentially also (see Table 1). We created 1,000 sets of 1,054 randomly positioned non-overlapping segments of equal size and probe density as those rare deletions observed in the meta-controls (see **Materials and Methods**). Brain-expressed genes were enriched among overlapping genes [*OR* = 1.67 (1.17–2.26)] and disrupted genes [*OR* = 2.08 (1.71–2.49)]; the enrichment for brain expressed and other brain genes sets was comparable to the enrichment in observed data.

**Table 1:** Properties of observed deletions in the meta-controls versus randomly placed simulated deletions.

	Meta-Controls (observed rare deletions)	Simulated Segments
**Segments**		
N	1054	1054
Median Length (kb)	51.1	51.1
Overlapping ≥1 Gene	412*	530 (498–557)
Disrupting ≥1 Gene	359*	472 (442–499)
**Overlapping Genes**		
N	538*	818 (739–900)
Gene length (median, kb)	65.7	57.1 (45.5–71.2)
>100 kb length genes	225*	311 (284–339)
>500 kb length genes	74	74 (62–87)
>1Mb length genes	29*	23 (17–29)
Brain-Expressed (OR)	1.63	1.51 (1.26–1.77)
Neuronal Activity (OR)	1.61	1.67 (1.17–2.26)
Synapse (OR)	2.04	1.98 (1.28–2.93)
Learning (OR)	1.78	1.71 (0.86–2.78)
**Disrupted Genes**		
N	345*	497 (462–532)
Gene length (median, kb)	162.9*	131.8 (115.9–152.1)
Brain-Expressed (OR)	2.04	2.08 (1.71–2.49)
Neuronal Activity (OR)	1.84	2.25 (1.50–3.07)
Synapse (OR)	2.33	2.85 (1.82–4.24)
Learning (OR)	2.84	2.25 (0.98–3.81)

Here we compare the observed properties for rare deletions in meta-controls to that of 1000 simulated random segments, matched for size and probe-density. For simulated random deletions for each property, we list the median value, and in parentheses we list the 95% range. We have noted values from observed deletions that deviate (*p*<0.05) from the simulations with asterisks (*). The first set of values compare observed rare deletions to random segments. The number and median length are the same since segments were matched for these properties. In the next two lines we list the numbers of overlapping and disrupted genes. The next set of values compare data on the set of genes overlapping observed deletions to simulated segments. We list the number of overlapping genes altogether, and exceeding specific gene length thresholds (100 kb, 500 kb, and 1 Mb). Finally we list the odds ratio for the three brain-function gene sets for the set of overlapping genes. The final set of values compare similar data on the set genes disrupted by rare deletions to simulated segments. Disrupted genes stratified by length is not shown, and is similar to that of overlapping genes.

However, there are two key differences in the results of real rare CNVs and simulated CNVs. Observed rare deletions overlap 35% fewer genes than random segments – suggesting unsurprisingly that deletions overlapping genes are selected against. Possibly, events affecting potentially critical genes that, if affected, disrupt normal human development are selected against. But, on the other hand, the pattern for the largest genes is strikingly different – the observed rare deletions actually overlap 26% more of those genes >1 Mb in length than random segments. This suggests a predilection for large genes that cannot be accounted for simply by their larger genomic footprint.

### Gene size, structure, and genic density all independently predict whether a gene is independently affected by a CNV

To explain the discrepancy between the size and number of genes affected in real CNVs and simulated segments, we speculated that while rare events affecting genes are negatively selected against, those that affect large genes might be less strongly selected against. Possibly, large genes have certain structural features that tend to make them relatively preferred targets of rare CNVs above and beyond their simple large size. For example, a CNV within a long gene might be more likely to fall within a large intron and not disrupt the coding sequence, and therefore have less-clear relevance to gene function. Furthermore, since genes tend not to overlap, a CNV of a particular size that overlaps larger genes may affect fewer genes than one that overlaps many smaller genes, and may therefore be less likely to impact some nearby essential gene.

To test whether these factors might play a role we tabulated three relevant structural features for each gene (see **Materials and Methods**): (1) transcript length, (2) a gene neighborhood density score, representing the expected number of additional nearby genes that a randomly placed CNV affects, and (3) a gene structure score, that represents the expectation that a randomly placed overlapping deletion is fully intronic. The first parameter simply accounts for the size of the ‘target’. The other two parameters account for the possibility that CNVs overlapping certain genes might be more likely to be functionally consequential. We found that all of these variables individually correlated with the likelihood that a gene is overlapped by deletions in meta-controls (see Figure S1). We then conducted a conditional analysis and found that even though they are inter-correlated, they each independently predict the probability that a gene is deleted in the meta-controls – removal of any single parameter significantly affects a logistic regression model's predictive ability (see Table 2). The additional factors of gene density and structure could account for the reduced number of affected genes overall and the increased proportion of larger genes compared to random segments in the genome.

**Table 2:** CNV-propensity score parameters.

	Multivariate Analysis		
Parameter	*β*		*p* (model)
Gene size (Mb)	2.40	(1.78–3.01)	1.2×10^−17^
Gene neighborhood density (genes/CNV)	−0.11	(−0.15–−0.07)	1.4×10^−7^
Gene structure score	1.31	(0.27–2.33)	0.019
Intercept	−3.31	(−3.46–−3.15)	

Each of the three parameters significantly improves the likelihood model predicting whether a gene overlaps a rare deletion within the meta-controls. We tested three parameters: (1) the **gene size**, which represents a straightforward parameter representing the length of the gene in Mb, (2) the **gene neighborhood density score** is the average number of additional genes that a CNV overlapping the gene overlaps, and (3) the **gene structure score** which is the proportion of CNVs overlapping a gene that is fully intronic. For each parameter we list the multivariate logistic regression beta and 95% confidence interval. We also list the statistical significance of the change in log-liklihood by removing the variable from the model.

One possible strategy to correct gene set based analyses is to devise a score that encapsulates the structural features of genes, and their predicted propensity to be affected by a CNV. This provides a robust approach to assess pathway enrichment in he suboptimal situation when controls are not available (e.g. when evaluating a collection of de novo case-only Autism deletions). We present such a CNV-propensity gene score (*CNVprop*) that represents an empirical estimate of the log-likelihood that a gene is overlapped by a CNV based on gene structural features based on the parameters from Table 2. *CNVprop* can be used as a covariate within a logistic regression framework in assessing enrichment of a gene set. We provide the *CNVprop* scores of genes in Table S6. While other methods to correct for gene size have been proposed in the literature, they do not specifically account for additional effects from gene density and intron structure, which are likely specific to CNV events. This approach, however, is still not ideal since it fails to account for multiple genes contributed by a single event, or genes being affected multiple times by an individual CNV event.

### Gene enrichment of brain function genes in schizophrenia case and control CNVs are equivalently significant

To further demonstrate application of gene set analysis and its potential pitfalls, we used a large data set published by the ISC with many rare (<1% frequency) deletions and duplications identified from 3,391 affected by schizophrenia and 3,181 unaffected individuals (see Table S3).

In order to replicate the analysis published by Walsh et al [1], we conducted set-based analyses of genes disrupted by CNV events within the ISC cases. We observed enrichment of brain-expressed genes (*p* = 3×10^−11^, two-tailed Fisher's exact, Figure 2). However, when we examined genes disrupted within *controls* in the ISC, we observed similar evidence for brain-expressed genes (*p* = 3×10^−10^, see Figure 2). Critically, the odds ratios (ORs) for enrichment of brain-expressed genes among genes disrupted in affected individuals and unaffected individuals were difficult to distinguish in this analysis. We observed similar trends towards enrichment for brain-expressed genes overlapped by CNVs (OR = 1.1, *p* = 0.06 for cases, OR = 1.08, *p* = 0.20 for controls, data not shown) and overlapped by very rare single event CNVs (OR = 1.5, *p* = 0.004 for cases, OR = 1.6, *p* = 0.01 for controls, data not-shown). Furthermore, with the exception of the learning genes, all brain function gene sets demonstrated significant enrichment within ISC cases and controls (see Figure 2).

We applied the same analysis to a data set with a small number of CNVs published by Walsh *et al* and demonstrated similar effects (see Figure 2). Cases tended to be more statistically significant for all of the gene sets than controls, since they were better powered with more affected genes. However, confidence intervals were wide in this analysis, and it was unclear it there were true case-control differences.

In both data sets – while statistically significant enrichment for brain function genes is observed in cases, it is not clear that the effect size is any different than in controls.

### Application of cnv-enrichment-test to previously published schizophrenia data sets

We applied the case-control *cnv-enrichment-test* to check CNVs published by the ISC and by Walsh et al to test whether case events were enriched for genes with brain function relative to controls. In the ISC data, we had already reported elsewhere increased genome-wide rates and sizes for case CNVs [2]. Walsh et al had demonstrated genome-wide enrichment separately.

We applied the *cnv-enrichment-test* to the four gene sets (*brain-expressed*, *neuronal-activity*, *learning* and *synapse* as described above). The results in Table 3 report the empirical 1-tailed p-values for a test of enrichment of the genes in the set relative to the genome-wide baseline rates of all CNVs; for the smaller gene sets standard asymptotic tests yielded unreliable estimates, due to the sparse nature of the data (for example 7 case events, 0 control events for neuronal genes in Walsh et al). In this context, the empirical significance values obtained via permutation will be robust to these sparse cell counts. Of course, for the larger gene sets, and all of the gene sets in the larger ISC data set, analytical *p*-values corresponded closely to permuted *p*-values.

**Table 3:** Assessing if brain-function genes increase schizophrenia risk in published CNV data sets.

Gene Set	ISC		Walsh et al	
	OR = (e*^γ^*)	*P* (empirical, 1 tailed)	OR = (e*^γ^*)	*P* (empirical, 1 tailed)
Brain-Expressed	1.03	0.19	1.11	0.41
Neuronal Activity	1.18	0.038	N/A	0.0004
Learning	1.38	0.0085	1.66	0.35
Synapse	0.97	0.58	3.35	0.12

We used the *cnv-enrichment-test* to test whether any of four brain function gene sets were enriched in two CNV published datasets. In the first column we list the tested gene set. In the second two columns we list results for the ISC data set, and in the final two columns we list results for the Walsh et al data set. For each data set we present the odds ratio for schizophrenia for each gene set and the one-tailed empirical p-value.

There was no evidence of enrichment among case-CNVs compared to control CNVs for *brain-expressed* and *synapse* genes (*p*>0.12, one-tailed analysis, see Table 3). This is in marked contrast to the observed enrichment of these same brain gene sets in the case-only analyses presented in Figure 2 that did not account for gene size.

However, the *neuronal-function* gene set demonstrated evidence of association to schizophrenia cases for both Walsh et al (*p* = 0.00045) and the ISC data (*p* = 0.04). There was also evidence of association of the *learning* gene set within the ISC data (*p* = 0.009) but not in the Walsh et al data (*p* = 0.35).

We want to emphasize that these results are not adjusted for multiple hypotheses testing – and the plausible number of independent gene sets. In this study alone we have tested four separate gene sets. Ultimately, convincing associations will require larger data sets. As additional samples are genotyped for CNVs, the relevance of the *neuronal-function* genes might be more clearly established.

Of note, considering only deletions within the ISC data, the effect of *neuronal-function* gene set enrichment is stronger (p = 0.0067, with higher rates in cases). Similarly, considering only deletions within the ISC data, the effect of the *learning* gene set enrichment is also stronger (p = 0.002, with higher rates in cases).

In both cases *neuronal-function* and *learning* gene sets, the effect sizes associated with an event affecting a gene is modest ranging from 1.2–1.7. This suggests that even if the set associations are ultimately validated, that rare CNV events affecting genes within these sets certainly do not fully explain the pathogenicity of rare CNVs.

### Conclusions

The *cnv-enrichment-test* is an extremely versatile test to identify whether a gene set of interest is associated with case-control status. We have shown that it is robust to confounders, such as case-control differences in CNV rate and gene size, while standard gene set enrichment approaches are not.

Since the *cnv-enrichment-test* can be applied easily to a wide range of gene sets, there may be the temptation to examine data sets by testing a compendium of gene-sets. Generally, we discourage this approach, and urge investigators to look at specific sets of interest. Assessing the significance of association statistics when testing a large compendium of gene-sets is complex since there is a large number of highly overlapping sets; correcting for the large burden of multiple hypotheses testing appropriately can be challenging. However, should one decide to test such a compendium of gene-sets, it is important that investigators permute the case-control status within their own data set, and apply the same battery of tests to make sure that the actual data set is obtaining levels of significance that are beyond that of the permuted data sets.

We have also shown that pathway analyses with standard gene set enrichment approaches are confounded by gene size and structure. This issue is of particular importance when considering genes with brain function – since those genes are significantly larger than other human genes. We have demonstrated how a large set of *brain-expressed* genes seem to be impacted by CNVs in both case and control populations when using gene set enrichment approaches, and how this effect is largely the consequence of the size of these genes. The *brain-expressed* genes were selected for having significantly greater expression in neuronal tissues as opposed to non-neuronal tissues. Certainly genes with important brain functions that are ubiquitously expressed in all tissues might be missed by such a strategy, as might genes with very low expression levels overall. However, we observed very similar results for three other separately curated sets of genes with brain function; this suggests that gene size and spurious pathway associations may be of particular importance for brain function genes.

The approach we describe here can be applied more broadly than within the context of CNVs; the *cnv-enrichment-test* can be applied to any situation where disease-associated genomic segments are defined. For example, linkage disequilibrium blocks around associated SNPs can be defined as disease-associated genomic-segments. The potential for gene size and structure confounding pathway analyses extends beyond CNV studies, and applies equally to pathway analyses within other types of genetic studies, including SNP association studies, as noted by Wang et al [27] and exon re-sequencing studies. For example, in a study looking at classes of genes that are disproportionately affected by rare exonic mutations, the total length of the coding sequence will be a key confounding variable. Similarly, studies looking at classes of genes that contain a single SNP nominally associated to disease, confounding variables might include the number of independent SNPs examined, the physical size of the gene, and the recombination hotspots across the length of the gene. In any case, careful case-control comparisons are essential to avoid these confounders.

Many of the genes involved in brain function are compelling candidate genes for neurological and psychiatric diseases – and indeed they may be the most vulnerable to CNVs. The purpose of this manuscript is not to question the results of the original publications, but to rather set up a rigorous statistical approach that allows investigators to accurately estimate effect sizes of events impacting specific gene sets of interest and also to precisely replicate reported results.

## Materials and Methods

### Compiling Gene Sets

#### Brain-Expressed

To identify genes with specific expression in the brain, we obtained a large publicly available human tissue expression microarray panel (GEO accession: GSE7307) [28]. We analyzed the data using the robust multi-array (RMA) method for background correction, normalization and polishing [29]. We filtered the data excluding probesets with either 100% ‘absent’ calls (MAS5.0 algorithm) across tissues, expression values <20 in all samples, or an expression range <100 across all tissues. To represent each gene, we selected the corresponding probeset with the greatest intensity across all samples. We included expression profiles from some 96 healthy tissues and excluded disease tissues and treated cell lines. We averaged expression values from replicated tissues averaged into a single value. To assess whether genes had differential expression for CNS tissues, we compared the 27 tissue profiles that represented brain or spinal cord to the remaining 69 tissue profiles with a one-tailed Mann-Whitney rank-sum test. We identified those genes obtaining *p*<0.01 as preferentially expressed.

#### Synapse

We downloaded Gene Ontology [30] structure and annotations on December 2006. Since it was available, we used a previous version of Gene Ontology to ensure independence from the results of recent genetic scans. We expanded human gene annotations to include annotations from orthologous genes, identified through Homologene [30] from model organisms. We identified those genes that were annotated with the ‘Synaptic Junction’ code (GO:0045202), or descendents of that code.

#### Neuronal Activity

We downloaded the list of genes within the category ‘Neuronal Activities’ (BP00166) listed in the Panther database [12].

#### Learning

We downloaded the list of genes within the category ‘Behavior-Learning’ listed in the Ingenuity Application.

To avoid spurious results and focus on a consistent set of genes across all studies, we included in our analysis only autosomal genes that (1) had at least one annotations in GO and (2) passed quality control criteria in the data set used to identify brain-expressed genes. The resulting set consisted of 14,565 annotated genes.

### Obtaining Walsh CNV data

We obtained rare event deletions and duplications from Table 2 in the original publication of the data [1].

### Obtaining ISC CNV data

Rare (<1% frequency) event deletions and duplications were provided directly by request from the International Schizophrenia Consortium.

### Identifying Rare CNVs in the Meta-controls

We obtained data from unaffected individuals with informed consent from four Institutional Review Board approved studies: macular degeneration [26], myocardial infarction [25], bipolar disease [8], and multiple sclerosis [24]. We obtained Affymetrix 6.0 raw intensity data for all samples and ran the Birdsuite software on each plate individually [31]; CNV calls were based on Birdseye output. We then analyzed healthy unaffected individuals from each of four studies separately. First we filtered individuals on SNP data, removing individuals with >5% missing data. Second, in situations where Birdseye called two nearby segments (<10 kb) with identical copy number and there was a low confident segment in between (LOD<3), we merged those segments. Third, we exclude all CNVs that (1) overlap CNVs from a map of common variation [32], or (2) failed stringent quality control criteria (<20 kb in length or <10 LOD or <10 probes). Fourth we removed those individuals in each study that were outliers in either excessive number of CNVs, or in excessive aggregate length of CNVs – we defined outlier as the median plus the 1.5 times the inter-quartile range. We then combined all CNVs into a single data set, and identified single-events (i.e. non-overlapping) deletions.

### Placing Genomic Segments

We produced 1000 sets of non-overlapping segments throughout the genome. Each set consisted of segments matched for size and probe-denisty (+/−10%) to each observed single-event deletions in meta-controls. Since we were simulating rare events, random events were not allowed to overlap regions with known copy number variation [32] or in regions where we observed an overlapping event (i.e. not a singleton) in the meta-controls.

### Defining Gene Parameters

For each gene we defined three parameters (1) gene length, (2) gene neighborhood density score, and (3) gene structure score.

Gene length was simply the length of the gene transcript in mega-basepairs.

To calculate a neighborhood density score for a gene, we consider a CNV overlapping a gene. The neighborhood density score is then the expected number of additional nearby genes overlapped by the same CNV. To empirically estimate the distribution of sizes of rare CNVs, we utilized the sizes, *s*, of observed single event deletions in the meta-controls. Then to calculate the gene neighborhood density score, *gd_i_*, for gene *i*, we used the following formula:
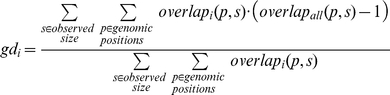
where *p* is a genomic position, *overlap_i_(p, s)* is an indicator function that is 1 if a segment of length *s* starting at position *p* overlaps gene *i*, or is otherwise 0. Similarly *overlap_all_(p, s)* is the number of genes that a segment of length *s* starting at position *p* overlaps. In the numerator we subtract one off, since we want to exclude gene *i* itself

To calculate a gene structure score, we calculated the expected proportion of overlapping CNVs that would not affect the coding sequence of the gene (i.e. be fully intronic). To empirically estimate the distribution of sizes of rare CNVs, we again used the sizes, *s*, of observed single event deletions in the meta-controls. Then to calculate the gene structure score, *gs_i_*, for gene *i*, we used the following formula:
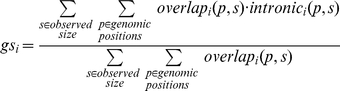
where *p* is a genomic position, *overlap_i_(p, s)* is an indicator function that is 1 if a segment of length *s* starting at position *p* overlaps gene *i*, or is otherwise 0. Similarly *intronic_i_(p, s)* is an indicator variable that is 1 if a segment of length *s* starting at position *p* does not overlap a coding sequence, or otherwise is 0.

### Statistical Models to Assess Gene Set Enrichment Across CNVs

In order to produce a framework to test gene-sets and their association to disease, we used a linear/logistic regression framework in which phenotype is regressed on the number of genes intersected (or disrupted) by one or more CNVs and covariates. We considered five different models to test for enrichment of CNVs in a pathway of interest, and tested them with simulated datasets.

For a disease outcome, a standard model, **M0**, is as follows:
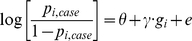
where for individual *i*, *g_i_* is the number of genes in a pathway of interest that intersected/disrupted by a CNV. The *θ* term is the logistic regression intercept and represents the background log likelihood for each individual, while *γ* is the logistic regression parameter for *g_i_*.

Model **M1** controls for potential genome-wide differences in CNV burden between cases and controls:
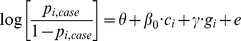
where *c_i_* is the total number of CNVs in a given individual *i*. The *β_0_* term is the logistic regression parameter for *c_i_*.

Model **M2** alternatively controls for CNV size:
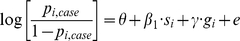
where *s_i_* is the individual's mean CNV size in kb. If for a particular individual *c_i_* = 0 (i.e. they do not have any CNVs) then *s_i_* is set to the sample mean of *s* rather than 0 or missing. (Otherwise, if many individuals have no CNVs, a strong correlation will be induced between the rate and average size of CNVs.) The *β_1_* term is the logistic regression parameter for *s_i_*.

Model **M3** alternatively controls for an individual's total CNV burden expressed in terms of total kb deleted or duplicated, written here as the product of c_i_ and *s*_i_:
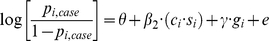
The *β_2_* term is the logistic regression parameter for (*c_i_*·*s_i_*).

Finally, the *cnv-enrichment-test* model controls explicitly for potential case/control differences in both the number and size distributions of CNVs:
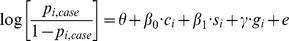
This is the model introduced in the main text labeled as the “*cnv-enrichment-test*”. As above, if for a particular individual *c_i_* = 0 (they do not have any CNVs) then *s_i_* is set to the mean size of all CNVs in the sample as opposed to zero.

Under all circumstances, the null hypothesis for the 2-sided test of enrichment is H_0_: *γ* = 0.

### Testing Models in a Simulated Case-Control Framework

We conducted simulations to understand the performance characteristics of these different analytic approaches (**M0** **–** **M3** and *cnv-enrichment-test*) to test for enrichment of case CNVs in a set of genes. We explicitly adopt extreme conditions in these simulations, to best illustrate the robustness of each approach under the broadest range of conditions.

For each individual, we simulated data for a single hypothetical chromosome, 250 Mb in length. We placed 2000 evenly-spaced, non-overlapping genes on the hypothetical chromosome, where every fifth gene was designated as a “brain gene”. We assigned brain genes to be considerably larger than other genes (50 kb versus 10 kb). In all scenarios we simulated CNV data for 2000 cases and 2000 controls. For cases and controls, the mean CNV size was either 60 kb or 100 kb, as detailed in Table S2 (range 10 kb to 150 kb, standard deviation 30 kb). Under all scenarios, individuals had either 0 or 1 CNV, with rates given in Table S2.

All datasets were simulated under the null hypothesis of no enrichment for brain genes; that is, CNVs were randomly placed on the hypothetical chromosome, similarly for both causal and controls. Under five scenarios, **S0** to **S4**, we altered the mean CNV rate and CNV size for cases and controls independently, in order to induce enrichment of CNVs in brain genes arising solely as a consequence of CNV rate and size. Under the first scenario, **S0**, there were no differences between cases and controls in the rate and size of CNVs: we therefore expected all methods to give appropriate type I error rates here. Under **S1**, the rate of CNVs was higher in cases. Under **S2**, the average CNV size was smaller in cases. Under **S3**, cases had a greater number, and larger, CNVs than controls. Under **S4**, cases had a greater number, but smaller, CNVs than controls.

For each scenario, we simulated 10,000 datasets to calculate the type I error rate for the enrichment test, for a nominal rate of 0.05.

### Implementation

This test is implemented in PLINK v1.07 (–cnv-enrichment-test). It is appropriate for either continuous or disease traits and allows for the inclusion of multiple other covariates and for empirical significance tests.

The following examples illustrate basic usage. If the file genes.dat contains the locations of all genes (i.e. as available from the resources section of the PLINK website, glist-hg18) and the file pathway.txt is a file of gene names forming the pathway to be tested for enrichment and the CNV data are in the files mycnv.cnv, mycnv.cnv.map and mycnv.fam (see website CNV page for details), then one can ask whether a) genes are enriched for CNVs, b) a subset of genes are enriched, relative to the whole genome, c) a subset of genes are enriched, relative to all genes. The latter form of the enrichment test might be desirable, for example, to determine whether any enrichment is general to all genes, or specific to a subset of genes.

***a) Enrichment of genic CNVs***

./plink ––cfile mycnv

––cnv-count genes.dat

––cnv-enrichment-test

***b) Enrichment of pathway genes CNVs, relative to all CNVs***

./plink ––cfile mycnv

––cnv-count genes.dat

––cnv-subset pathway.txt

––cnv-enrichment-test

***c) Enrichment of pathway genes CNVs, relative to all genic CNVs***

./plink ––cfile mycnv

––cnv-intersect genes.dat

––cnv-write my-genic-cnv

./plink ––cfile my-genic-cnv

––cnv-count genes.dat

––cnv-subset pathway.txt

––cnv-enrichment-test

The usual modifiers (to define intersection differently, allow for a certain kb border around each gene, filter on CNV size, type or frequency, etc) are all available. Under all circumstances, 2-sided asymptotic p-values are returned. Alternatively, permutation testing can be applied and 1-sided empirical p-values are returned (positive enrichment in cases, based on estimated regression coefficient).

For additional information consult the PLINK website (http://pngu.mgh.harvard.edu/purcell/plink/), the resources subsection (gene list) (http://pngu.mgh.harvard.edu/purcell/plink/res.shtml), or the CNV file format subsection (http://pngu.mgh.harvard.edu/purcell/plink/cnv.shtml).

## Supporting Information

Figure S1 Features predicting whether a gene overlaps a CNV in the meta-controls. A. Here we plot the distribution of the genes that are not deleted (*n* = 14,027, blue) and the genes that are deleted (*n* = 538, red) separately for the meta-controls. Deleted genes are larger with a median of 66 kb compared to genes not deleted with a median of 27 kb. Medians and inter-quartile ranges are indicated with the boxes, while the range indicates the 2.5 to 97.5 percentiles for both distributions. B. We plot the fraction intrinic fraction score as a function of gene size. Larger genes tend to have potentially greater proportions of events that could be fully intronic. Red points indicate deleted genes while blue point indicate the remainder. C. Here we plot the local gene density, i.e., the number of other nearby genes overlapped by a CNV as a function of gene size. Events overlapping large genes tend not to overlap other nearby genes. Red points indicate deleted genes while blue points indicate the remainder.
(1.85 MB TIF)

Table S1 Gene size of brain genes highlighted in three CNV-association studies. Here we list affected genes within gene sets highlighted in three neuropsychiatric disease studies. In the first column we list the study, in the next two columns we list the functional gene sets and their source. In the fourth and fifth column we list the genes, and their sizes. In the final column we list the mean size. Many of the genes highlighted in all three studies are very large genes. *For the Walsh et al. study, these genes were compiled from multiple brain function gene sets.
(0.09 MB DOC)

Table S2 Simulated distribution of CNV rate and size in cases and controls. We tested different statistical models as outlined in Materials and Methods (M0–M4) for false positive associations under each of five extreme scenarios (S0–S4) outlined in the above table. For a single hypothetical chromosome, 250Mb in length, we placed 2000 evenly-spaced, non-overlapping genes. Every fifth gene was designated as a “brain gene”; brain genes were set to be considerably larger than other genes (50kb versus 10kb). In all scenarios we simulated CNV data for 2000 cases and 2000 controls, specifying the mean CNV size was either 60kb or 100kb (range 10kb to 150kb, standard deviation 30kb) and CNV rate per individual. Under the first scenario, S0, there were no differences between cases and controls in the rate and size of CNVs: we therefore expected all methods to give appropriate type I error rates here. Under S1, the rate of CNVs was higher in cases. Under S2, the average CNV size was smaller in cases. Under S3, cases had a greater number, and larger, CNVs than controls. Under S4, cases had a greater number, but smaller, CNVs than controls.
(0.04 MB DOC)

Table S3 Collections examined in this study. Our study examined affected and unaffected individuals from the ISC, Walsh *et al.*, and Zhang *et al.*. We also used unaffected populations from four separate studies (meta-controls). For each study we list the number of samples, the genotyping technology used to identify CNVs (Representational Oligonucleotide Microarray Analysis (ROMA), Affymetrix 5.0 (5.0) or Affymetrix 6.0 (6.0)), the number of observed events, how we defined a ‘rare’ event, their size, and the number of genes affected by those events.
(0.06 MB DOC)

Table S4 Deletion in meta-controls events called in four separate populations. Meta-control rare deletion events were called based on Affymetrix 6.0 arrays. For each of the four collections we list the number of samples, the number of rare deletions >20 kb and the ratio of deletions to samples, the number of rare deletions >100 kb and the ratio of deletions to samples, and finally the median event size.
(0.04 MB DOC)

Table S5 Gene ontology codes with the largest genes are enriched in meta-controls. Here we list 13 GO codes with an average gene length >200 kb and their descriptions in the first four columns. In the next three columns we list the number of genes for each code overlapping rare deletions in meta-controls, the odds ratio, and the statistical significance. All enrichment analyses *p*-values are calculated with Fisher's exact test; enrichment is calculated for both disrupted and deleted genes. In the final three columns we list the number of genes disrupted by rare deletions in meta-controls, the odds ratio, and the statistical significance.
(0.06 MB DOC)

Table S6 CNVprop scores of genes.
(0.66 MB TXT)
