# Supplementary figures and images for: Accurately Assessing the Risk of Schizophrenia Conferred by Rare Copy-Number Variation Affecting Genes with Brain Function
Source: PLoS Genet. 2010 Sep 9;6(9):e1001097. doi: 10.1371/journal.pgen.1001097 (PMC2936523; doi:10.1371/journal.pgen.1001097)

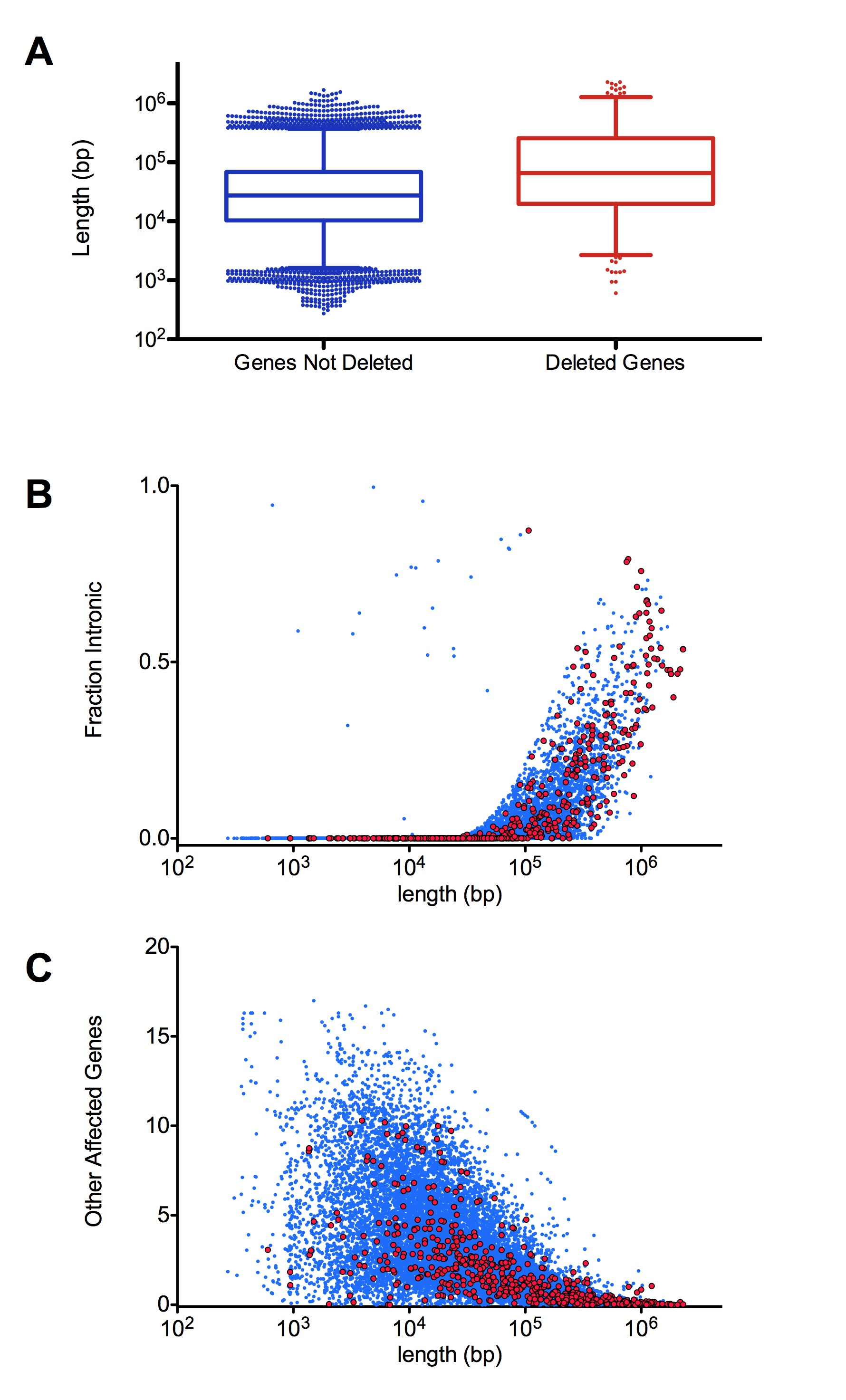

Supplement: Figure S1 — Features predicting whether a gene overlaps a CNV in the meta-controls. A. Here we plot the distribution of the genes that are not deleted (n = 14,027, blue) and the genes that are deleted (n = 538, red) separately for the meta-controls. Deleted genes are larger with a median of 66 kb compared to genes not deleted with a median of 27 kb. Medians and inter-quartile ranges are indicated with the boxes, while the range indicates the 2.5 to 97.5 percentiles for both distributions. B. We plot the fraction intrinic fraction score as a function of gene size. Larger genes tend to have potentially greater proportions of events that could be fully intronic. Red points indicate deleted genes while blue point indicate the remainder. C. Here we plot the local gene density, i.e., the number of other nearby genes overlapped by a CNV as a function of gene size. Events overlapping large genes tend not to overlap other nearby genes. Red points indicate deleted genes while blue points indicate the remainder. (1.85 MB TIF) [file pgen.1001097.s001.tif]
