# Supplementary material for: Accurately Assessing the Risk of Schizophrenia Conferred by Rare Copy-Number Variation Affecting Genes with Brain Function
Source: PLoS Genet. 2010 Sep 9;6(9):e1001097. doi: 10.1371/journal.pgen.1001097 (PMC2936523; doi:10.1371/journal.pgen.1001097)
Supplement: Table S1 — Gene size of brain genes highlighted in three CNV-association studies. Here we list affected genes within gene sets highlighted in three neuropsychiatric disease studies. In the first column we list the study, in the next two columns we list the functional gene sets and their source. In the fourth and fifth column we list the genes, and their sizes. In the final column we list the mean size. Many of the genes highlighted in all three studies are very large genes. *For the Walsh et al. study, these genes were compiled from multiple brain function gene sets. (0.09 MB DOC) [file pgen.1001097.s002.doc]

***Supplementary Table 1.***

| **Study** | **Pathway** | **Source** | **Genes** | **Size (kbp)** | **Average Gene Size (kbp)** |
| --- | --- | --- | --- | --- | --- |
| Walsh et al *Science* 2008 (schizophrenia) | Neuro-developmental Pathways* | Ingenuity / Panther | *DLG2* | 2,172 | 701 |
|  |  |  | *MAGI2* | 1,437 |  |
|  |  |  | *ERBB4* | 1,163 |  |
|  |  |  | *GRM7* | 880 |  |
|  |  |  | *PTPRM* | 840 |  |
|  |  |  | *PTK2* | 343 |  |
|  |  |  | *PRKAG2* | 321 |  |
|  |  |  | *CAV1* | 271 |  |
|  |  |  | *LAMA1* | 176 |  |
|  |  |  | *SLC1A3* | 82 |  |
|  |  |  | *PRKCD* | 32 |  |
| Zhang et al *Molecular Psychiatry* 2008 (bipolar) | Behavior (learning) | Ingenuity | *PTPRD* | 2,298 | 474 |
|  |  |  | *PARK2* | 1,380 |  |
|  |  |  | *GRM7* | 880 |  |
|  | Psychological Disorders | Ingenuity | *CNTNAP2* | 2,305 |  |
|  |  |  | *NRXN1* | 1,112 |  |
|  |  |  | *APP* | 290 |  |
|  |  |  | *RCAN2* | 271 |  |
|  |  |  | *PI4KA* | 151 |  |
|  |  |  | *MED15* | 80 |  |
|  |  |  | *GNB1L* | 67 |  |
|  |  |  | *SNAP29* | 31 |  |
|  |  |  | *UFD1L* | 29 |  |
|  |  |  | *COMT* | 27 |  |
|  |  |  | *RTN4R* | 27 |  |
|  |  |  | *ZDHHC8* | 16 |  |
|  |  |  | *ADH1B* | 15 |  |
|  |  |  | *ZNF74* | 14 |  |
|  |  |  | *TXNIP* | 4 |  |
|  |  |  | *CLDN5* | 2 |  |
| Glessner et al *Nature* 2009 (autism) | Neuronal Cell Adhesion | Gene Ontology | *NRXN1* | 1,112 | 716 |
|  |  |  | *ASTN2* | 990 |  |
|  |  |  | *CNTN4* | 957 |  |
|  |  |  | *NLGN1* | 885 |  |
|  | Ubiquitin | Uniprot | *PARK2* | 1,380 |  |
|  |  |  | *RFWD2* | 262 |  |
|  |  |  | *UBE3A* | 102 |  |
|  |  |  | *FBXO40* | 37 |  |

***Supplementary Table 1. Gene size of brain genes highlighted in three CNV-association studies.*** Here we list affected genes within gene sets highlighted in three neuropsychiatric disease studies. In the first column we list the study, in the next two columns we list the functional gene sets and their source. In the fourth and fifth column we list the genes, and their sizes. In the final column we list the mean size. Many of the genes highlighted in all three studies are very large genes. *For the Walsh et al study, these genes were compiled from multiple brain function gene sets.
