# Supplementary material for: Accurately Assessing the Risk of Schizophrenia Conferred by Rare Copy-Number Variation Affecting Genes with Brain Function
Source: PLoS Genet. 2010 Sep 9;6(9):e1001097. doi: 10.1371/journal.pgen.1001097 (PMC2936523; doi:10.1371/journal.pgen.1001097)
Supplement: Table S2 — Simulated distribution of CNV rate and size in cases and controls. We tested different statistical models as outlined in Materials and Methods (M0–M4) for false positive associations under each of five extreme scenarios (S0–S4) outlined in the above table. For a single hypothetical chromosome, 250Mb in length, we placed 2000 evenly-spaced, non-overlapping genes. Every fifth gene was designated as a “brain gene”; brain genes were set to be considerably larger than other genes (50kb versus 10kb). In all scenarios we simulated CNV data for 2000 cases and 2000 controls, specifying the mean CNV size was either 60kb or 100kb (range 10kb to 150kb, standard deviation 30kb) and CNV rate per individual. Under the first scenario, S0, there were no differences between cases and controls in the rate and size of CNVs: we therefore expected all methods to give appropriate type I error rates here. Under S1, the rate of CNVs was higher in cases. Under S2, the average CNV size was smaller in cases. Under S3, cases had a greater number, and larger, CNVs than controls. Under S4, cases had a greater number, but smaller, CNVs than controls. (0.04 MB DOC) [file pgen.1001097.s003.doc]

***Supplementary Table 2.***

|  | **Cases** | | **Controls** | |
| --- | --- | --- | --- | --- |
| **Scenario** | **CNV rate** | **Mean size (kb)** | **CNV rate** | **Mean size (kb)** |
| S0 | 0.25 | 100 | 0.25 | 100 |
| S1 | 0.25 | 100 | 0.05 | 100 |
| S2 | 0.25 | 60 | 0.25 | 100 |
| S3 | 0.25 | 100 | 0.05 | 60 |
| S4 | 0.25 | 60 | 0.05 | 100 |

***Supplementary Table 2. Simulated distribution of CNV rate and size in cases and controls.*** We tested different statistical models as outlined in the **Supplementary Note** (**M0-M4**) for false positive associations under each of five extreme scenarios (**S0-S4**) outlined in the above table. For a single hypothetical chromosome, 250Mb in length, we placed 2000 evenly-spaced, non-overlapping genes. Every fifth gene was designated as a “brain gene”; brain genes were set to be considerably larger than other genes (50kb versus 10kb). In all scenarios we simulated CNV data for 2000 cases and 2000 controls, specifying the mean CNV size was either 60kb or 100kb (range 10kb to 150kb, standard deviation 30kb) and CNV rate per individual. Under the first scenario, **S0**, there were no differences between cases and controls in the rate and size of CNVs: we therefore expected all methods to give appropriate type I error rates here. Under **S1**, the rate of CNVs was higher in cases. Under **S2**, the average CNV size was smaller in cases. Under **S3**, cases had a greater number, and larger, CNVs than controls. Under **S4**, cases had a greater number, but smaller, CNVs than controls.
