# Supplementary material for: Accurately Assessing the Risk of Schizophrenia Conferred by Rare Copy-Number Variation Affecting Genes with Brain Function
Source: PLoS Genet. 2010 Sep 9;6(9):e1001097. doi: 10.1371/journal.pgen.1001097 (PMC2936523; doi:10.1371/journal.pgen.1001097)
Supplement: Table S3 — Collections examined in this study. Our study examined affected and unaffected individuals from the ISC, Walsh et al., and Zhang et al.. We also used unaffected populations from four separate studies (meta-controls). For each study we list the number of samples, the genotyping technology used to identify CNVs (Representational Oligonucleotide Microarray Analysis (ROMA), Affymetrix 5.0 (5.0) or Affymetrix 6.0 (6.0)), the number of observed events, how we defined a ‘rare’ event, their size, and the number of genes affected by those events. (0.06 MB DOC) [file pgen.1001097.s004.doc]

***Supplementary Table 3.***

| Study |  | **Walsh et al** | | **Zhang et al** | | **International Schizophrenia Consortium** | | **Meta-Controls** |
| --- | --- | --- | --- | --- | --- | --- | --- | --- |
| Description |  | Cases | Controls | Cases | Controls | Cases | Controls |  |
|  |  |  |  |  |  |  |  |  |
| # Samples |  | 150 | 268 | 1001 | 1034 | 3391 | 3181 | 2415 |
| Genotyping |  | ROMA | ROMA | 6.0 | 6.0 | 5.0/6.0 | 5.0/6.0 | 6.0 |
| Event Type |  | Del/Dup | Del/Dup | Del | Del | Del/Dup | Del/Dup | Del |
| Definition |  | Singleton | | Singleton | | <1% | | Singleton |
| # >20 kb Events | |  |  |  |  |  |  | 1054 |
| # >100 kb Events | | 21 | 12 | 175 | 135 | 3602 | 3151 | 244 |
|  |  |  |  |  |  |  |  |  |
| Event Size (kb) | Median | 461.9 | 410.0 | 163.9 | 161.1 | 182.9 | 181.1 | 51.1 |
|  |  |  |  |  |  |  |  |  |
| Affected Genes | Disrupted | 21 | 9 | 59 | 48 | 871 | 713 | 345 |
|  | Overlapping | 138 | 40 | 179 | 160 | 2,709 | 2,052 | 538 |

***Supplementary Table 3. Collections examined in this study.*** Our study examined affected and unaffected individuals from the ISC, Walsh et al, and Zhang et al. We also used unaffected populations from four separate studies (meta-controls). For each study we list the number of samples, the genotyping technology used to identify CNVs (Representational Oligonucleotide Microarray Analysis (ROMA), Affymetrix 5.0 (5.0) or Affymetrix 6.0 (6.0)), the number of observed events, how we defined a ‘rare’ event, their size, and the number of genes affected by those events.
