# Supplementary material for: Accurately Assessing the Risk of Schizophrenia Conferred by Rare Copy-Number Variation Affecting Genes with Brain Function
Source: PLoS Genet. 2010 Sep 9;6(9):e1001097. doi: 10.1371/journal.pgen.1001097 (PMC2936523; doi:10.1371/journal.pgen.1001097)
Supplement: Table S4 — Deletion in meta-controls events called in four separate populations. Meta-control rare deletion events were called based on Affymetrix 6.0 arrays. For each of the four collections we list the number of samples, the number of rare deletions >20 kb and the ratio of deletions to samples, the number of rare deletions >100 kb and the ratio of deletions to samples, and finally the median event size. (0.04 MB DOC) [file pgen.1001097.s005.doc]

***Supplementary Table 4.***

| **Study** | **MIGEN** | **AMD** | **GAIN** | **MS** |
| --- | --- | --- | --- | --- |
| Description | Early Myocardial infarction Study | Age-Related Macular Degeneration Study | GAIN Bipolar Disorder Study | Multiple Sclerosis Study |
|  |  |  |  |  |
| # Samples | 866 | 454 | 850 | 245 |
|  |  |  |  |  |
| >20 kb deletions | 400 | 175 | 372 | 107 |
| >20 kb Dels/Individual | 0.462 | 0.385 | 0.438 | 0.437 |
|  |  |  |  |  |
| >100 kb deletions | 88 | 36 | 99 | 21 |
| >100 kb Dels/Individual | 0.102 | 0.079 | 0.116 | 0.086 |
|  |  |  |  |  |
| Median Event Size (kb) | 50.8 | 47.7 | 52.5 | 48.7 |

***Supplementary Table 4. Deletion in meta-controls events called in four separate population.*** Meta-control rare deletion events were called based on Affymetrix 6.0 arrays. For each of the four collections we list the number of samples, the number of rare deletions > 20 kb and the ratio of deletions to samples, the number of rare deletions >100 kb and the ratio of deletions to samples, and finally the median event size.
