# Supplementary material for: Accurately Assessing the Risk of Schizophrenia Conferred by Rare Copy-Number Variation Affecting Genes with Brain Function
Source: PLoS Genet. 2010 Sep 9;6(9):e1001097. doi: 10.1371/journal.pgen.1001097 (PMC2936523; doi:10.1371/journal.pgen.1001097)
Supplement: Table S5 — Gene ontology codes with the largest genes are enriched in meta-controls. Here we list 13 GO codes with an average gene length >200 kb and their descriptions in the first four columns. In the next three columns we list the number of genes for each code overlapping rare deletions in meta-controls, the odds ratio, and the statistical significance. All enrichment analyses p-values are calculated with Fisher's exact test; enrichment is calculated for both disrupted and deleted genes. In the final three columns we list the number of genes disrupted by rare deletions in meta-controls, the odds ratio, and the statistical significance. (0.06 MB DOC) [file pgen.1001097.s006.doc]

***Supplementary Table 5.***

| **GO Code** | **Function** | ***N*** | **Mean Gene Size (kb)** | **Genes Overlapping Singleton Deletion** | | | **Genes Disrupted by Singleton Deletion** | | |
| --- | --- | --- | --- | --- | --- | --- | --- | --- | --- |
|  |  |  |  | *N* | OR | *p* | *N* | OR | *p* |
| GO:0008066 | glutamate receptor activity | 36 | 285 | 4 | 3.3 | 0.043 | 4 | 5.1 | 0.010 |
| GO:0007215 | glutamate signaling pathway | 25 | 281 | 2 | 2.3 | 0.24 | 2 | 3.6 | 0.12 |
| GO:0008038 | neuron recognition | 34 | 264 | 4 | 3.5 | 0.036 | 4 | 5.5 | 0.0085 |
| GO:0005913 | cell-cell adherens junction | 37 | 256 | 3 | 2.3 | 0.16 | 3 | 3.6 | 0.058 |
| GO:0044456 | synapse part | 111 | 250 | 11 | 2.9 | 0.0029 | 8 | 3.2 | 0.0054 |
| GO:0004112 | cyclic-nucleotide phosphodiesterase activity | 25 | 242 | 2 | 2.3 | 0.24 | 2 | 3.6 | 0.12 |
| GO:0008037 | cell recognition | 48 | 223 | 4 | 2.4 | 0.10 | 4 | 3.7 | 0.027 |
| GO:0005605 | basal lamina | 34 | 221 | 2 | 1.6 | 0.36 | 2 | 2.6 | 0.19 |
| GO:0030165 | PDZ domain binding | 44 | 218 | 4 | 2.6 | 0.080 | 3 | 3.0 | 0.087 |
| GO:0045211 | postsynaptic membrane | 94 | 215 | 8 | 2.4 | 0.024 | 5 | 2.3 | 0.075 |
| GO:0045202 | synapse | 209 | 209 | 15 | 2.0 | 0.015 | 11 | 2.3 | 0.019 |
| GO:0050839 | cell adhesion molecule binding | 26 | 204 | 2 | 2.2 | 0.25 | 2 | 3.4 | 0.13 |
| GO:0050769 | positive regulation of neurogenesis | 28 | 202 | 1 | 1.0 | 1.0 | 1 | 1.5 | 0.49 |

***Supplementary Table 5. Gene ontology codes with the largest genes are enriched in meta-controls.*** Here we list 13 GO codes with an average gene length > 200 kb and their descriptions in the first four columns. In the next three columns we list the number of genes for each code overlapping rare deletions in meta-controls, the odds ratio, and the statistical significance. All enrichment analyses p-values are calculated with Fisher’s exact test; enrichment is calculated for both disrupted and deleted genes. In the final three columns we list the number of genes disrupted by rare deletions in meta-controls, the odds ratio, and the statistical significance.
